# Navigating an evolving microbial landscape: emerging antimicrobial resistance trends and precision stewardship in Tianjin tertiary hospitals (2021–2023)

**DOI:** 10.3389/fcimb.2025.1629038

**Published:** 2025-09-08

**Authors:** Yong-li Wan, Tao Han, Qiang Sun, Donghao Wang, Jun Li, Li-jie Wang, Min Peng, Yin Li, Qing-guo Feng, Chun-guang Liu, Jie Xu, Bin Bao, Mei Su, Zhi-yong Fei, Xu-liang Wang, Xiao-bo Liu

**Affiliations:** ^1^ Intensive Care Unit, National Health Commission (NHC) Key Laboratory of Hormones and Development, Chu Hsien-I Memorial Hospital and Tianjin Institute of Endocrinology, Tianjin Medical University, Tianjin, China; ^2^ Tianjin Key Laboratory of Metabolic Diseases, Tianjin Medical University, Tianjin, China; ^3^ Intensive Care Unit, Tianjin Medical University Cancer Institute and Hospital, National Clinical Research Center for Cancer, Key Laboratory of Cancer Prevention and Therapy, Tianjin, Tianjin’s Clinical Research Center for Cancer, Tianjin, China; ^4^ Intensive Care Unit, Tianjin Union Medical Center, The First Affiliated Hospital of Nankai University, Tianjin, China; ^5^ Geriatrics Department, First Teaching Hospital of Tianjin University of Traditional Chinese Medicine, Tianjin, China; ^6^ Intensive Care Unit, Tianjin Medical University General Hospital, Tianjin, China; ^7^ Intensive Care Unit, Tianjin First Center Hospital, Tianjin, China; ^8^ Intensive Care Unit, Tianjin Fifth Central Hospital, Tianjin, China; ^9^ Intensive Care Unit, Tianjin Baodi Hospital, Tianjin, China; ^10^ Intensive Care Unit, Tianjin Economic–Technological Development Area (TEDA) Hospital, Tianjin, China; ^11^ Intensive Care Unit, Tianjin Xiqing Hospital, Tianjin, China; ^12^ Intensive Care Unit, Tianjin Hongqiao Hospital, Tianjin, China; ^13^ Intensive Care Unit, Haibin People’s Hospital of Tianjin Binhai New Area, Tianjin, China; ^14^ Intensive Care Unit, Tianjin Jinnan Hospital, Tianjin, China; ^15^ Intensive Care Unit, Tianjin Peking University Medical Ocean Oil Hospital, Tianjin, China

**Keywords:** AMR, multicenter studies, tertiary care, *Escherichia coli*, infection control

## Abstract

**Objective:**

To evaluate microbial distribution and antimicrobial resistance (AMR) patterns in clinical isolates from 13 tertiary hospitals and one secondary hospital in Tianjin (2021–2023) to inform precision-driven antimicrobial stewardship and infection control interventions.

**Methods:**

In this retrospective, multicenter study, we collected routine diagnostic specimens—including sputum, fecal samples, secretions, blood, and drainage fluids. Data were processed per standardized protocols (CARSS, CHINET) and interpreted using current CLSI-M100 breakpoints. Statistical analyses were performed with SPSS 20.0 (significance set at two‐tailed *P* < 0.05).

**Results:**

Sputum specimens increased from 39.1% to 43.0%, while urine samples and secretions declined. *Klebsiella pneumoniae* prevalence rose from 18.3% to 20.3%, whereas *Escherichia coli* remained stable. *E. coli* maintained excellent susceptibility to carbapenems and amikacin (≤2% resistance); notably, ceftazidime/avibactam resistance declined from 7.2% to 3.4% (*P* = 0.005) amid a significant increase in cefepime resistance (24.4% to 29.6%, *P* < 0.001). *K. pneumoniae* exhibited parallel trends, with escalating resistance to β-lactam/β-lactamase inhibitor agents. In *Pseudomonas aeruginosa*, aminoglycoside, and carbapenem profiles remained stable, while ceftazidime/avibactam sensitivity markedly improved, suggesting shifts in underlying resistance mechanisms. *Acinetobacter baumannii* showed enhanced susceptibility to aminoglycosides, β-lactam inhibitors, and fluoroquinolones; however, carbapenem-resistant isolates continued to exhibit near-universal resistance. Among gram-positive pathogens, methicillin-resistant *Staphylococcus aureus* sustained near-universal β-lactam resistance with improved rifampicin sensitivity, while glycopeptides and linezolid remained fully active. *Enterococcus faecalis* demonstrated reduced ampicillin resistance, contrasting with *E. faecium*’s near-pan-resistance to β-lactams and fluoroquinolones.

**Conclusion:**

Evolving, species-specific AMR patterns in Tianjin hospitals highlight the urgent need for real-time, regionally stratified surveillance and molecularly informed stewardship strategies to guide targeted antimicrobial interventions and improve clinical outcomes.

## Introduction

Antimicrobial resistance (AMR) in healthcare settings has emerged as one of modern medicine’s most formidable challenges (Jones, 2001). In hospitals, nosocomial infections by multidrug-resistant organisms complicate patient management and lead to prolonged hospital stays, increased mortality, and substantial economic burdens (Gan et al., 2012; Ramirez et al., 2020). In China, rising levels of antibiotic resistance have been extensively documented by national surveillance programs such as CARSS and CHINET (Wang, 2022). However, considerable regional variability exists, and local data are crucial for tailoring empiric therapy and optimizing infection control measures (Tolera et al., 2018).

Tianjin, one of China’s major metropolitan areas, has witnessed significant clinical and demographic shifts over recent years that may influence pathogen distribution and resistance patterns. Despite the availability of nationwide data, there remains a paucity of comprehensive, regional studies examining the temporal dynamics of microbial profiles and corresponding antibiotic resistance trends within Tianjin hospitals. Such analyses are essential, as changes in specimen types, patient case mix, and antibiotic prescribing practices can profoundly affect the local epidemiology of infectious agents.

In this study, we retrospectively analyzed microbiological data from 14 major hospitals in Tianjin over three years (2021–2023), 13 of which are tertiary hospitals. Our objectives were to investigate the evolving distribution of clinical specimens and pathogen species—including key subgroups such as gram-negative and gram-positive bacteria, Enterobacteriaceae, and non-fermenters—and to assess their resistance profiles against commonly used antimicrobial agents. Special emphasis was placed on tracking the resistance trends of predominant pathogens such as *Escherichia coli*, *Klebsiella pneumoniae*, *Acinetobacter baumannii*, *Pseudomonas aeruginosa*, and methicillin-resistant *Staphylococcus aureus* (MRSA).

By comparing resistance rates and microbial compositions over time and across institutions, we aim to provide critical insights for clinicians in Tianjin, facilitating the rational use of antibiotics and strengthening local infection control strategies. In addition, our study offers a valuable reference for regional and national AMR surveillance efforts, ultimately contributing to developing more precise antibiotic stewardship programs.

## Methods

### Study design and setting

This retrospective, multicenter study was conducted among 14 hospitals (13 tertiary and one secondary hospital) in Tianjin over three years from 2021 to 2023. The study aimed to evaluate the microbial distribution and AMR patterns in clinical isolates, providing essential data to optimize antibiotic usage and infection control measures in the region.

### Ethical approval

This study was not required for ethical approval since it was based on legally notifiable, anonymized surveillance data collected for public health purposes. All data were handled strictly with data confidentiality agreements and relevant institutional guidelines.

### Data collection

Microbiological data were sourced from the clinical laboratories of 14 Tianjin hospitals participating in the National AMR Monitoring System (CARSS). Data spanning 2021 to 2023 were downloaded directly from CARSS. This cohort comprises one specialized hospital and 13 comprehensive hospitals—please refer to the author affiliations for the complete list. Each hospital provided comprehensive records of routinely collected diagnostic specimens and the corresponding bacterial identification and antimicrobial susceptibility testing results. These data were aggregated according to protocols prescribed by national surveillance systems (e.g., CARSS and CHINET). All participating centers adhered to standardized laboratory methods for bacterial isolation, identification, and susceptibility testing.

### Data analysis

Surveillance data spanning January 2021 to December 2023 were collected under protocols compliant with institutional review board guidelines and confidentiality agreements endorsed by the Anti-Cancer Association. Data processing followed the big data analysis standards recommended by the National Bacterial Resistance Monitoring Network (Ju et al., 2023). Following the first-isolate-per-patient principle, duplicate bacterial isolates from the same patient and species were systematically excluded to prevent sampling bias. A domestic, self-developed drug sensitivity big data statistical analysis system, integrated with WHONET software, was used for synchronous two-way data verification. Records were excluded if the filling rate for a given drug susceptibility test was <70% or if the test coverage for a single strain was <90%. Antimicrobial susceptibility testing and interpretation followed the latest CLSI-M100 Antimicrobial Susceptibility Testing Standard breakpoints.

Recognizing that, despite a standardized network, slight variations in laboratory protocols and patient populations across the participating institutions might exist, we conducted extensive sensitivity analyses to adjust for potential inter-center variability. These measures ensure that any biases in the dataset are minimized, thus bolstering the robustness and validity of our statistical conclusions. Furthermore, including one specialized hospital alongside 13 comprehensive hospitals enhances the representation of diverse clinical settings, which undoubtedly contributes to the external validity of our study.

Statistical analyses were conducted using SPSS 20.0 (IBM Corp.). Categorical variables, including resistance rates and pathogen distributions, were analyzed using chi-square (χ²) tests or Fisher’s exact tests for small-sample comparisons, with two-tailed *P<*0.05 considered statistically significant. Temporal trends in resistance rates and inter-hospital variability were evaluated across the study period. Subgroup analyses stratified pathogens into clinically relevant categories: gram-negative bacteria, gram-positive bacteria, Enterobacteriaceae, and non-fermenters, enabling granular assessment of resistance dynamics. Data visualizations, including statistical plots, were generated using R software (version 4.2.1) with the ggplot2 package.

## Results

### Specimen distribution

**Figure 1:**
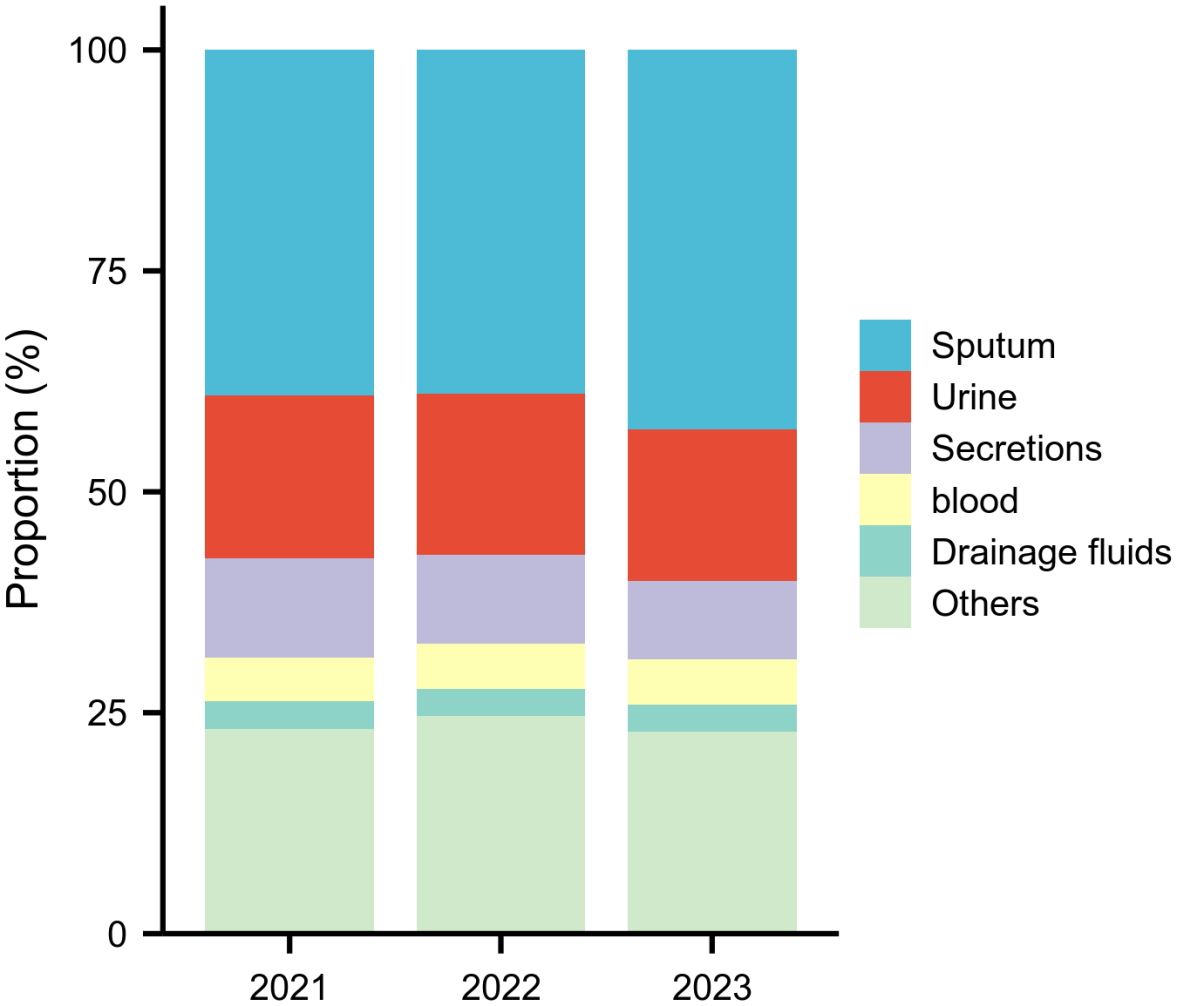
Annual Top Five Specimen Types (2021–2023) This figure shows the proportional distribution of the five most common specimen types, revealing a significant increase in sputum specimens and a decline in secretions over the three‑year period.

Surveillance data demonstrated significant shifts in specimen type distributions (**Figure 1**, **Supplementary Table S1**). The proportion of sputum specimens increased substantially from 39.1% (19,074 isolates) in 2021 to 43.0% (28,832 isolates) in 2023, while urine samples declined from 18.4% (8,991) to 17.1%, and secretions decreased from 11.2% (5,436) to 8.8%. Blood (5.0%→5.1%) and drainage fluids (3.1%→3.1%) maintained stable representation across the study period.

### Microbial distribution

#### Overall isolate composition

**Figure 2:**
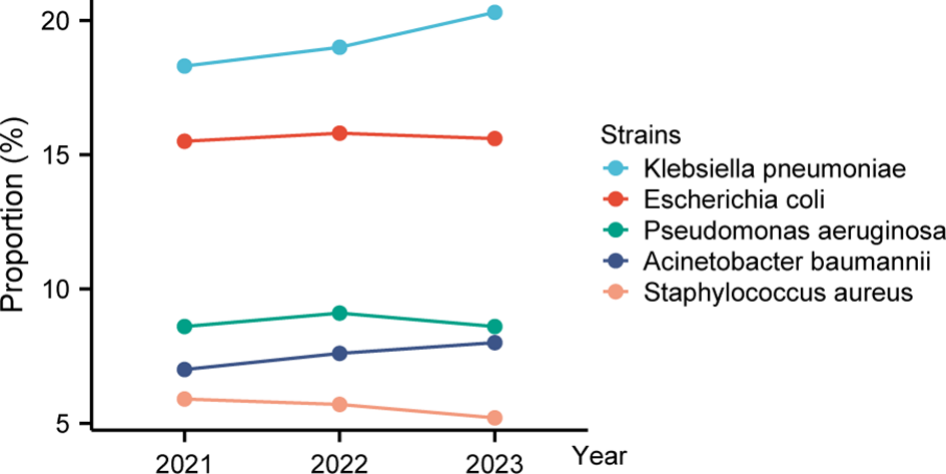
Trends in relative proportions of top five bacterial isolates (2021–2023). This line chart depicts how the relative proportions of the top five bacterial isolates have changed over three years. The top two—*Klebsiella pneumoniae* increased steadily, while *Escherichia coli* remained stable.

Between 2021 and 2023, the bacterial profile shifted noticeably. In 2021, the top pathogens were *K. pneumoniae* (18.3%), *E. coli* (15.5%), *P. aeruginosa* (8.6%), *A. baumannii* (7.0%), and *S. aureus* (5.9%) (**Figure 2**; **Supplementary Table S2**). By 2023, *K. pneumoniae* had risen to 20.3% as the dominant isolate, *A. baumannii* increased modestly to 8.0%, *E. coli* remained stable, and *S. aureus* declined to 5.2%.

#### Gram-positive pathogens

**Figure 3:**
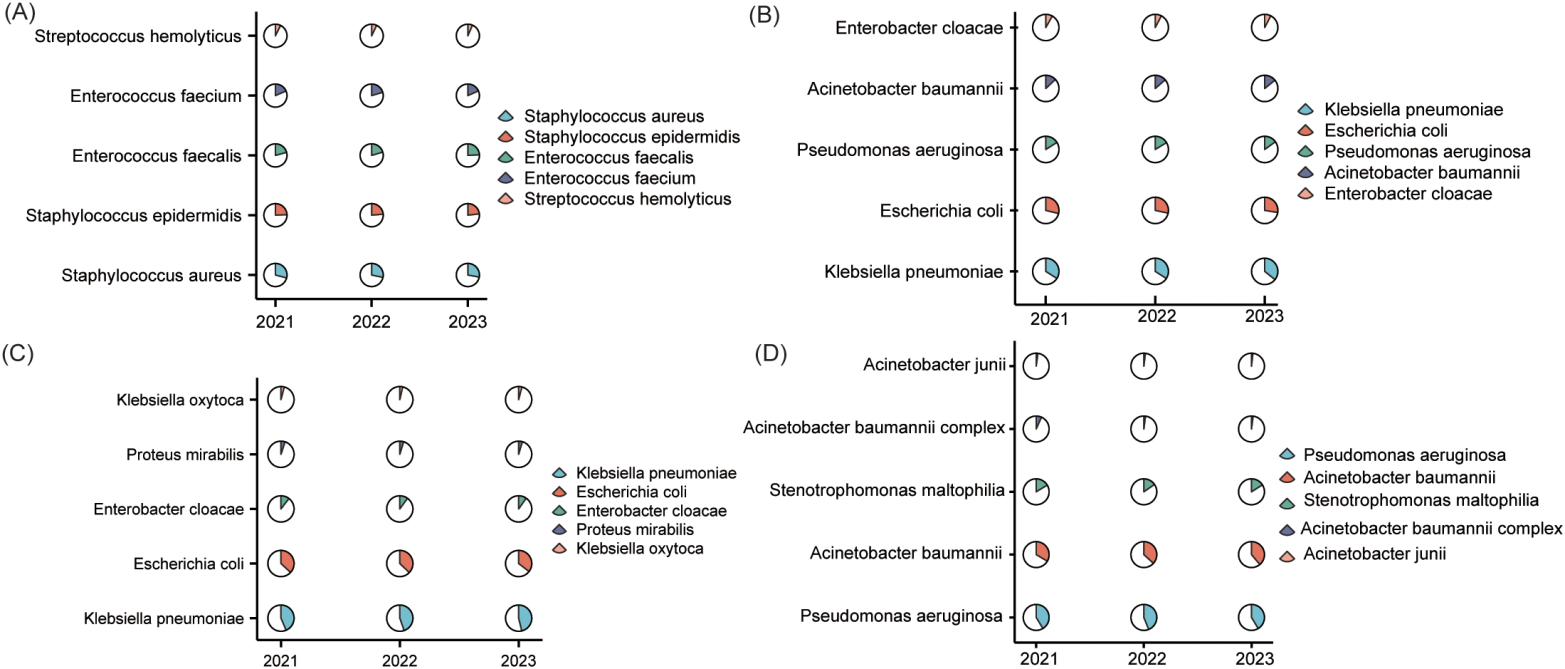
Taxonomic distribution of clinical pathogens (2021–2023). This composite figure presents the top five isolates for **(A)** gram-positive bacteria; **(B)** gram-negative bacteria; **(C)** Enterobacteriaceae; **(D)** Non-fermentative bacteria.

gram-positive profiles remained largely stable with subtle shifts (**Supplementary Table S3**; **Figure 3A**)*. S. aureus maintained predominance, decrea*sing slightly from 21.1% (2,852 isolates) in 2021 to 20.3% (3,480) in 2023, while *S. epidermidis* declined from 17.6% to 16.9%. *Enterococcus faecium increased* from 15.1% (2,041) to 17.6% (3,021). *Enterococcus faecalis* peaked at 15.2% in 2022 (2,112) before returning to 13.4% in 2023. Other gram-positive cocci remained a minor, consistent group.

#### Gram-negative pathogens

Among the top five gram-negatives, *K. pneumoniae* demonstrated a steady rise in prevalence from 25.3% (8,904) in 2021 to 27.3% (13,653) in 2023 (**Supplementary Table S4**; **Figure 3B**). *E. coli* maintained a stable proportional representation at approximately 21% despite rising absolute counts (7,551 to 10,466), while *P. aeruginosa* and *A. baumannii* exhibited minor proportional fluctuations(11.9%→11.5% and 9.6%→10.8%, respectively). *Enterobacter cloacae* consistently accounted for approximately 6% of cases throughout the study period.

#### Enterobacteriaceae and non-fermenters

Within Enterobacteriaceae, *K. pneumoniae*’s share climbed from 37.7% (8,904) in 2021 to 40.4% (13,653) in 2023, with *E. coli* remaining secondary (~31%) (**Supplementary Table S5**; **Figure 3C**). Minor taxa—*E. cloacae*, *S. marcescens*, and *P. mirabilis*—showed minimal proportional shifts. Conversely, non-fermenters consolidated into a duopoly; *P. aeruginosa* peaked at 40.3% in 2022, settling at 37.1%, while *A. baumannii* increased from 30.5% to 34.9% (**Supplementary Table S6**; **Figure 3D**). *S. maltophilia* remained consistently represented (14.9%→14.3%), with other species collectively below 2%.

### AMR patterns

Enterobacteriaceae, non-fermentative gram-negative bacteria, and gram-positive cocci—to enable straightforward cross-species comparisons and identify actionable insights. Enterobacteriaceae exhibit β-lactamase-mediated resistance while supporting species-specific interventions. Non-fermentative gram-negatives, often implicated in device-related infections, primarily rely on efflux pump and porin modifications, critical challenges in intensive care. In contrast, gram-positive pathogens highlight differing mechanisms, with MRSA’s β-lactam resistance versus enterococci’s adaptive vancomycin tolerance reflecting distinct hospital and community pressures. This framework, aligned with CLSI breakpoints and WHO priority lists, remains clinically relevant for pathogens linked to over 80% of AMR-related mortality.

#### Escherichia coli

**Figure 4:**
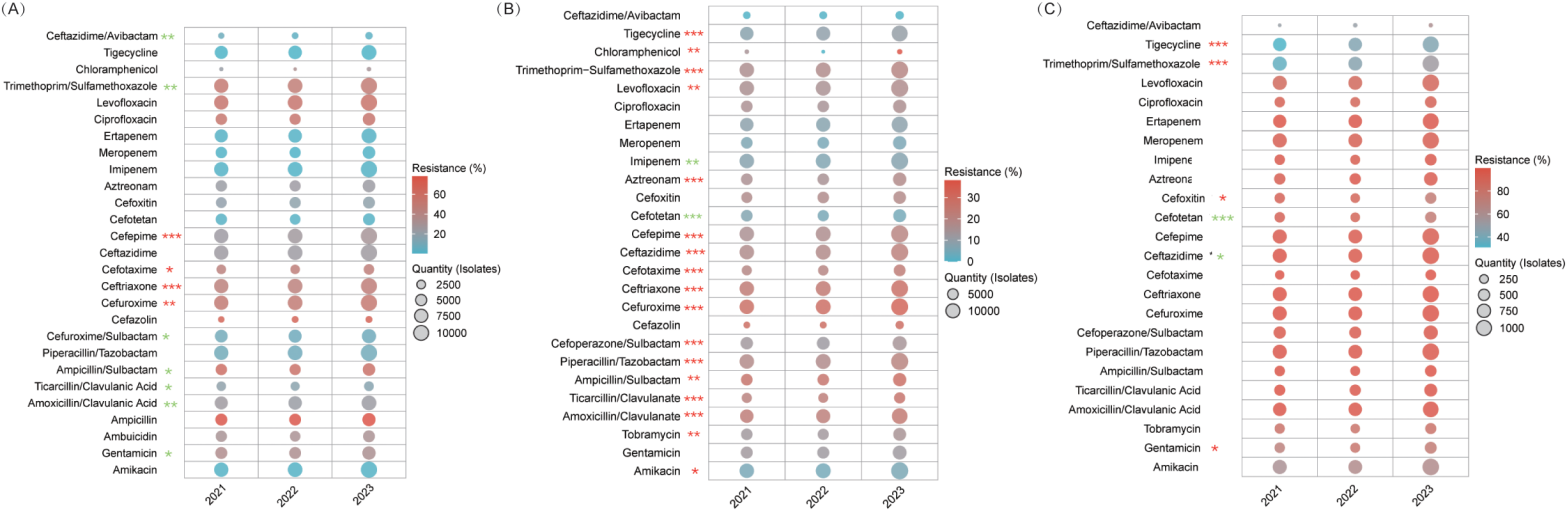
AMR in Enterobacteriaceae. This figure illustrates resistance trends among Enterobacteriaceae. **(A)** *E. coli*. **(B)** *Klebsiella pneumoniae*. **(C)** CRKP. * *P<*0.05, ** *P<*0.01, *** *P<*0.001. Red stars denote an increasing trend in resistance, while green stars indicate a declining trend.

Carbapenems, amikacin, and tigecycline consistently exhibited low resistance rates (atest whereas ceftazidime/avibactamie resistance significantly declined from 7.2% to 3.4% (*P*=0.005) compared to cefepime, which increased from 24.4% to 29.6% (*P*<0.001). Among β-lactamase inhibitor combinations, resistance for ticarcillin/clavulanate fell from 19.1% to 15.8% (*P=*0.015), while that for amoxicillin/clavulanate fluctuated between 23.0–25.5% (*P=*0.005). Third-generation cephalosporins showed increasing resistance (ceftriaxone: 44.5% to 47.2%; cefotaxime: 43.6% to 46.5%), and fluoroquinolones remained alarmingly high (53.1–56.0%), with levofloxacin rising slightly (54.4% to 56.0%, *P=*0.040). Piperacillin/tazobactam and cefoperazone/sulbactam retained moderate efficacy (≤9.2%) (**Figure 4A**, **Supplementary Table S7**).

#### Klebsiella pneumoniae

Carbapenems, amikacin, and ceftazidime/avibactam remained highly active (imipenem: 6.7–7.8%; meropenem: 5.3–6.3%; ceftazidime/avibactam: 0.4–0.5%). In contrast, resistance increased for piperacillin/tazobactam (18.2% to 20.4%) and ticarcillin/clavulanate (20.8% to 26.3%, both *P<*0.001), while cefepime surged from 15.6% to 20.0%. Cefotetan’s resistance declined (6.8% to 4.8%), tigecycline efficacy lessened (8.0% to 10.3%), and fluoroquinolone resistance remained steady (ciprofloxacin: 15.4–16.1%, *P=*0.087) (**Figure 4B**, **Supplementary Table S8**).

##### Carbapenem-resistant *Klebsiella pneumoniae*

CRKP exhibited near-universal resistance to β-lactams and carbapenems (imipenem: 94.3–94.8%; meropenem: 95.4–97.2%; third-generation cephalosporins: ≥97%). Aminoglycoside utility was limited, with amikacin’s sensitivity plateauing at 37–41% (*P=*0.174) and gentamicin resistance rising from 69.8% to 74.8% (*P=*0.037). Notably, cefotetan resistance dropped from 92.1% to 74.9% (*P<*0.001), boosting sensitivity threefold to 25.1%. Tigecycline and trimethoprim-sulfamethoxazole resistance significantly increased (31.2%→43.5% and 35%→53.1%, respectively; *P<*0.001), while ceftazidime/avibactam data remained limited (**Figure 4C**, **Supplementary Table S9**).

#### Pseudomonas aeruginosa

**Figure 5:**
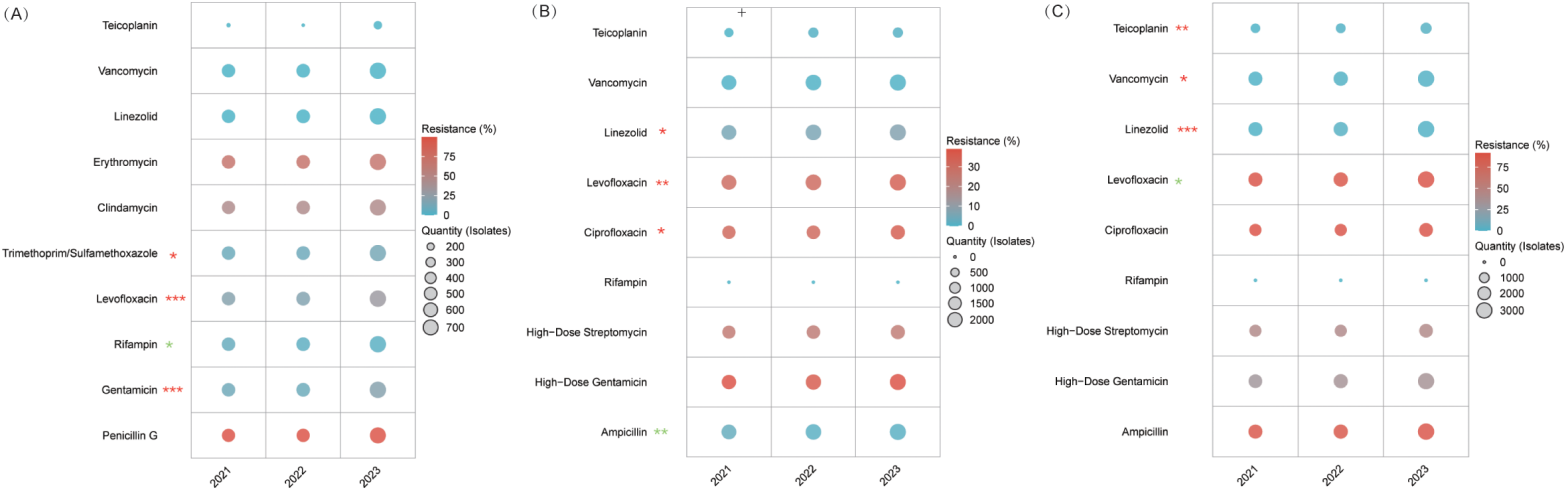
Resistance trends in MRSA and Enterococcus species. **(A)** MRSA. **(B)** *E*. *faecalis*. **(C)** *E*. *faecium*. Levofloxacin resistance decreased from 90.6% in 2021 to 88.6% in 2022, then rose to 90.2% in 2023 (*P* = 0.048), and linezolid resistance rose to 2.4% in 2022 before falling to 1.3% in 2023 (*P* < 0.001). Despite these significant trends, the 2021 and 2023 rates did not differ significantly, indicating overall stability in resistance.* *P<*0.05, ** *P<*0.01, *** *P<*0.001. Red stars denote an increasing resistance trend for those antibiotics, while green stars indicate a declining trend.

Aminoglycosides (amikacin 2.7–3.0%, tobramycin 3.9–4.6%, gentamicin 5.0–6.6%) and carbapenems (imipenem 17.4–17.9%, meropenem 19.5–20.0%) maintained stable resistance (P>0.05). Remarkably, ceftazidime/avibactam resistance improved from 98.0% to 87.6% (*P<*0.001), increasing sensitivity from 2.0% to 12.4%. Cefepime fluctuated (10.7–12.5%, *P=*0.034), fluoroquinolones remained effective (ciprofloxacin: 9.8–11.3%, *P=*0.053), and polymyxin B continued to perform well (resistance 2.0–7.5%) (**Figure 5A**, **Supplementary Table S10**).

##### Carbapenem-resistant *Pseudomonas aeruginosa*

**Figure 6:**
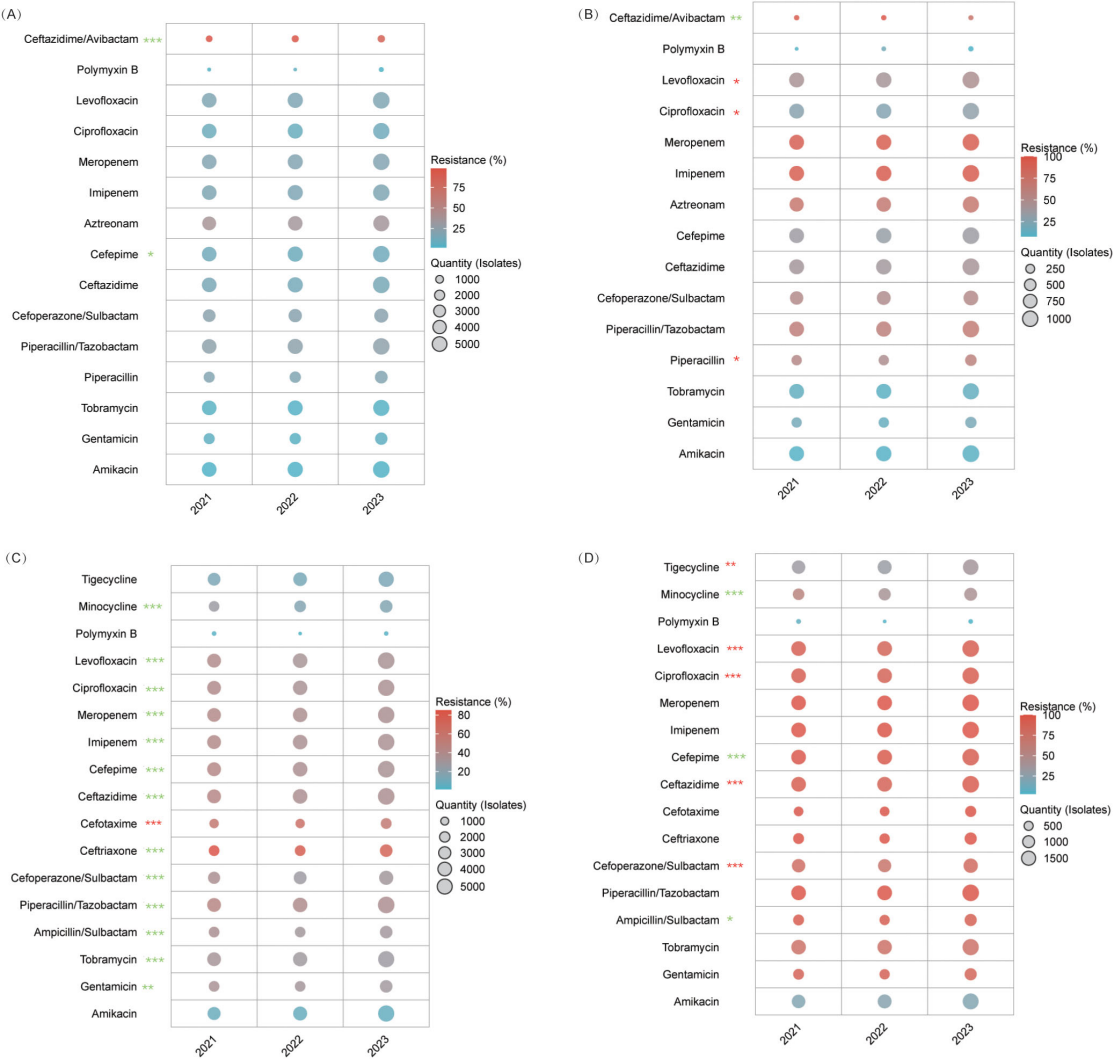
AMR Trends in *Pseudomonas aeruginosa* and *Acinetobacter* spp. **(A)** Carbapenem-susceptible *P. aeruginosa*. **(B)** CRPA. **(C)** *Acinetobacter baumannii*. Although the overall resistance trend for cefotaxime was statistically significant (*P*<0.001), its rate spiked from 55.9% to 62.5% before declining to 55%, with no significant difference observed between 2021 and 2023. **(D)** CRAB. Cefoperazone/sulbactam resistance was 79%, 71.2%, and 81.1%, and ceftazidime was 93.1%, 88.5%, and 94.4% for 2021, 2022, and 2023, respectively. Although the trends were significant (*P*<0.001), both agents dropped initially before rebounding, leaving 2021 and 2023 rates statistically similar.* *P<*0.05, ** *P<*0.01, *** *P<*0.001. Red stars indicate an increasing resistance trend for that antibiotic, while green stars indicate a declining trend.

CRPA maintained high carbapenem resistance (imipenem: 93.6–94.6%, meropenem: 91.8–93.5%) and aztreonam (67.6–70.2%). Aminoglycoside resistance varied moderately (amikacin 9.7–10.9%, tobramycin 11.0–14.0%), while fluoroquinolone resistance worsened (ciprofloxacin: 25.2%→30.5%, *P=*0.037; levofloxacin: 41.6%→47.2%, *P=*0.048). Conversely, ceftazidime/avibactam reduced resistance by 23.5% (from 96.2% to 72.7%; *P*=0.003), corresponding to an approximately 7-fold sensitivity increase, reaching 27.3%. Polymyxin B remained active (8.0–18.8%) (**Figure 6B**, **Supplementary Table S11**).

#### Acinetobacter baumannii

For *A. baumannii*, aminoglycosides improved—gentamicin resistance declined from 33.9% to 28.9% (*P<*0.001), and amikacin saw a slight decrease (8.1%→6.9%). β-lactamase inhibitor combinations also recovered, with ampicillin/sulbactam resistance dropping from 36.2% to 29.8% and piperacillin/tazobactam from 44.0% to 37.3% (*P<*0.001). Cefoperazone/sulbactam initially decreased from 36.6% to 27.2% before stabilizing at 30.0% (*P<*0.001). Fluoroquinolone resistance improved (ciprofloxacin: 39.8%→35.2%, levofloxacin: 39.6%→34.1%; *P<*0.001 for both), minocycline declined from 26.3% to 16.3% (*P<*0.001), and polymyxin B remained highly effective (1.2–3.5%). Cefoperazone/sulbactam showed emerging potential, achieving 72.8% sensitivity in 2022 (*P<*0.001) (**Figure 6C**, **Supplementary Table S12**).

##### Carbapenem-resistant *Acinetobacter baumannii*

CRAB isolates were nearly uniformly resistant to carbapenems (imipenem: 99.1–99.6%; meropenem: 99.8–100%) and witnessed increasing fluoroquinolone resistance (ciprofloxacin: 87.4–93.3%, levofloxacin: 86.9–92.3%; *P<*0.001). Amikacin maintained moderate activity (20.2–22.5%, *P=*0.285), while gentamicin (≥86%) and tobramycin (≥74%) were largely ineffective. Polymyxin B remained the most effective option (2.9–7.7%), minocycline resistance fell from 55.5% to 42.5% (*P<*0.001), and tigecycline resistance increased from 31.3% to 37.0% (*P=*0.004) (**Figure 6D**, **Supplementary Table S13**).

#### MRSA

MRSA maintained near-universal β-lactam resistance (penicillin G: 99–99.8%; *P=*0.201). Rifampicin improved markedly—resistance dropped from 7.7% to 3.7% (*P=*0.01)—while resistance to levofloxacin increased from 20.2% to 30.7% (*P<*0.001) and TMP-SMX from 8.9% to 13.6% (*P=*0.024). Gentamicin resistance nearly doubled (10.6%→20.5%, *P<*0.001), whereas clindamycin and erythromycin remained consistently high. In contrast, linezolid, vancomycin, and teicoplanin maintained 100% susceptibility, underscoring their status as therapeutic anchors (**Figure 5A**, **Supplementary Table S14**).

#### Enterococcus faecalis and Enterococcus faecium

*E. faecalis* (**Figure 5B**, **Supplementary Table S15**) and *E. faecium* (**Figure 5C**, **Supplementary Table S16**) displayed distinct resistance trends despite shared treatment strategies. In *E. faecalis*, ampicillin resistance significantly declined from 2.8% to 1.5% (*P=*0.001), and vancomycin and teicoplanin remained virtually effective (<1% resistance). However, fluoroquinolone resistance increased (ciprofloxacin: 31.9%→35.3%, *P=*0.044; levofloxacin: 30.2%→34.7%, *P=*0.002), and linezolid resistance rose from 5.6% to 7.5% (*P=*0.03), with gentamicin resistance remaining stable (36.2–39%, *P=*0.119). Conversely, E. faecium demonstrated near-pan-resistance to β-lactams (ampicillin: 88.0–89.5%, P=0.236) and fluoroquinolones (ciprofloxacin: 89.6–91.7%; levofloxacin: 88.6–90.6%), while its linezolid resistance remained low and relatively stable over the three years (1.0%, 2.4%, and 1.3%). In *E. faecium* isolates, glycopeptides maintained excellent efficacy with resistance rates ≤0.9%, while these isolates exhibited moderate resistance to aminoglycosides.

## Discussion

Our retrospective multicenter study, encompassing data from 14 tertiary hospitals in Tianjin over 2021–2023, provides a comprehensive snapshot of microbial distribution and antibiotic resistance trends in this region. Overall, our findings reveal significant shifts in specimen collection practices and the dynamics of pathogen prevalence and antimicrobial susceptibility patterns. These data are instrumental in tailoring local antibiotic stewardship and infection control policies.

### Post-pandemic specimen shifts and pathogen trends

One of the most striking trends noted in our study is the increasing proportion of sputum specimens, which rose from 39.1% in 2021 to 43.0% in 2023. This redistribution highlights intensified respiratory specimen surveillance, potentially indicating evolving diagnostic practices for pulmonary infections or modified hospital admission patterns during post-pandemic recovery phases (Simpson et al., 2003; Shi et al., 2023; Kim et al., 2025). In contrast, the proportions of other specimen types—such as fecal samples and secretions—remained relatively stable or decreased slightly, suggesting that improvements in collection protocols and diagnostic criteria may influence the overall specimen profile. These shifts underscore the need for the laboratory and clinical teams to continuously optimize specimen processing and ensure that diagnostic methods remain robust, particularly for high-risk samples (Mahale et al., 2024; Yang et al., 2024).

Regarding pathogen distribution, our data confirm the dominance of gram-negative bacteria in the clinical setting, with *Klebsiella pneumoniae* and *Escherichia coli* consistently representing the majority of isolates. Notably, the rising proportions of *K. pneumoniae* (from 18.3% in 2021 to 20.3% in 2023) and the stabilization of *E. coli* around 15–16% highlight a concerning trend. These changes parallel global trends reported in national surveillance networks such as CARSS and CHINET (Ashour and El-Sharif, 2009; Wan et al., 2017; Wei et al., 2019), although our data suggest that local epidemiology in Tianjin may exhibit unique characteristics possibly influenced by regional antibiotic usage practices and infection control measures. Notably, the relative stability of gram-positive pathogens, including *Staphylococcus aureus* and coagulase-negative staphylococci, indicates that while these organisms remain clinically significant, the major antimicrobial challenge in our setting continues to stem from gram-negative organisms.

### Evolving resistance dynamics and therapeutic implications

#### i. E. coli resistance: stable foundations, emerging challenges

Our multicenter surveillance of *E. coli* reveals a dual pattern: stable “therapeutic anchors” alongside emerging threats. Carbapenems and amikacin maintain resistance rates at ≤2%, confirming their role as first-line agents for critical infections (Zhang et al., 2013). Notably, ceftazidime/avibactam resistance dropped from 7.2% to 3.4%, highlighting its promise as a carbapenem-sparing option against carbapenemase producers.

Cephalosporin resistance is highly agent-dependent. For example, cefepime resistance increased significantly (from 24.4% to 29.6%, *P<*0.001), likely due to plasmid-borne CMY-2 type AmpC that hydrolyzes cefepime effectively, while cefotetan remains very low (≤2.3%) owing to its 7−methoxy structure, which confers stability against AmpC (Park et al., 2012). Similarly, incremental resistance rises in third-generation cephalosporins (ceftriaxone and cefotaxime) mirror global trends driven by CTX−M−15 Extended−Spectrum Beta−Lactamase—whose hydrolytic efficiency is three times higher than that of CTX−M−14—supporting current Infectious Diseases Society of America guidelines against their empirical use (Park et al., 2012; Taneja et al., 2012). Beta−lactam/β−lactamase inhibitor combinations show mixed results: ticarcillin/clavulanate resistance has declined, whereas amoxicillin/clavulanate fluctuates, suggesting regional differences in beta−lactamase variants (Taneja et al., 2012). Meanwhile, fluoroquinolone resistance consistently exceeds 50%, precluding their use as monotherapy (Park et al., 2012). We recommend screening for bla_DHA−1, an AmpC subtype, and qnrS, a plasmid−mediated fluoroquinolone resistance gene, to further characterize local resistance mechanisms.

These findings lead to three strategic imperatives for antimicrobial stewardship. First, integrating rapid molecular diagnostics with carbapenems and amikacin can maximize clinical efficacy while minimizing ecological impact. Second, precision prescribing must account for intra-class differences; for example, cefotetan’s sustained low resistance makes it preferable to cefepime. Third, the improved profile of ceftazidime/avibactam offers a viable strategy to reduce carbapenem overuse. Additional data showing a modest decline in gentamicin resistance and the maintained activity of piperacillin/tazobactam and cefoperazone/sulbactam for moderate infections further underscore the need for tailored data-driven therapy. In summary, tiered therapeutic escalation—reserving novel agents for critical cases and using conventional β-lactams in lower-acuity settings—combined with evolving surveillance and rapid diagnostics will be essential to preempt and counter emerging resistance mechanisms such as AmpC hyperproduction.

#### ii. Klebsiella pneumoniae: conventional susceptibility and the challenge of carbapenem resistance

Our analysis marks a critical turning point in the management of *Klebsiella pneumoniae*. Carbapenems such as meropenem and imipenem continue to play a vital role in non-carbapenem-resistant infections, with resistance rates hovering around 6–7%. Chinese multicenter data from 2016 to 2020 show meropenem susceptibilities of 94.2–96.1%, a testament to rapid real-time PCR detection of bla_KPC/bla_NDM and restrictive prescribing practices (Ju et al., 2023). In contrast, cefepime resistance has increased by roughly 28%, driven mainly by plasmid-mediated AmpC hyperproduction (Cho et al., 2015). Molecularly, the transposon-mediated transfer of carbapenemase genes like bla_OXA−48 is about five times less efficient than that of bla_AmpC, which helps delay resistance spread (Bonnin et al., 2025). Additionally, AmpC-overexpressing strains—such as *Enterobacter cloacae*—gain a competitive advantage during cephalosporin exposure, promoting pathogen replacement in clinical settings (Kalantar-Neyestanaki et al., 2015). Consequently, carbapenem-sparing strategies should avoid excessive cephalosporin use, favor non−cephalosporin agents (e.g., ampicillin-sulbactam), and incorporate quantitative PCR monitoring of bla_CMY/TEM copy numbers.

With beta-lactam/inhibitor combinations, trends diverge: ticarcillin/clavulanate resistance has risen due to inhibitor-resistant TEM variants (Pitout and Laupland, 2008), while amoxicillin/clavulanate remains stable; notably, cefotetan resistance has dropped by 29%. Ceftazidime/avibactam continues to show excellent activity (≤0.5% resistance) against key carbapenemase producers—even as tigecycline and fluoroquinolone resistance signal a need for caution. These findings support a rational therapeutic hierarchy: reserve carbapenems and ceftazidime/avibactam for life-threatening infections; use cefotetan and amikacin for moderate cases; and consider piperacillin/tazobactam in settings with high local susceptibility. Real-time resistance analytics and rapid diagnostics are essential for dynamically adjusting treatment strategies as further research unveils the molecular drivers behind cephalosporin and glycylcycline resistance.

CRKP exemplifies the multidrug resistance crisis, with over 95% resistance to both carbapenems and β-lactam/β-lactamase inhibitor combinations—a direct consequence of the widespread dissemination of bla_KPC and bla_NDM genes (Chen et al., 2021; Ge et al., 2024). This near-near-pan-resistant profile mirrors global trends and renders traditional agents such as piperacillin/tazobactam and third-generation cephalosporins largely ineffective (Lu et al., 2023). Despite this, cefotetan shows promise; its resistance has dropped by approximately 19%, and sensitivity has increased threefold to around 25%. This rebound, akin to reversals seen in *Enterobacter cloacae* after reduced cephalosporin use, suggests that lowering cephamycin pressure may partially restore susceptibility. Meanwhile, aminoglycoside efficacy is declining: gentamicin resistance continues to rise, and amikacin sensitivity remains unacceptably low (37–41%). Alarmingly, last-resort agents such as tigecycline and trimethoprim-sulfamethoxazole are rapidly losing effectiveness, emphasizing the need to restrict their use in vulnerable patients.

Clinically, these data call for a precision approach to combination regimens. Cefotetan’s resurgence supports its use as a backbone when combined with agents like high-dose tigecycline or siderophore cephalosporins. Limited data on ceftazidime/avibactam and declining tigecycline efficacy further underscore the necessity of real-time resistance monitoring and rapid molecular diagnostics—to reliably differentiate between KPC and metallo-β-lactamase producers. While CRKP’s resistance is formidable, strategic antibiotic cycling and focused stewardship can reclaim older agents and slow resistance progression. In an era of a stagnant antibiotic pipeline, global audits of antimicrobial use are crucial for minimizing collateral damage from past practices and ensuring that stewardship evolves with microbial adaptation.

#### iii. P. aeruginosa: a dual reality of susceptibility and resistance

Our data reveal a clear divergence based on carbapenem susceptibility. In carbapenem-susceptible isolates, aminoglycosides (amikacin and tobramycin) remain robust—with resistance consistently below 4.6%—and carbapenems (imipenem at ~17.7% and meropenem near 20%) retain efficacy, reflecting preserved porin (OprD) function and moderated efflux activity (Quale et al., 2006). In contrast, CRPA exhibits carbapenem resistance exceeding 91.8% due to OprD loss, MexAB-OprM hyperexpression, and AmpC overproduction, rendering these agents ineffective (Shu et al., 2017; Yang et al., 2025).

Cephalosporin profiles further distinguish these groups. Carbapenem-susceptible strains show moderate cefepime resistance (approximately 10–13%) and stable ceftazidime levels (~14–15%). For CRPA, while aztreonam resistance remains high (~68–70%), ceftazidime/avibactam resistance drops by roughly 23.5%—likely reflecting clonal shifts toward AmpC-derepressed, metallo-β-lactamase(MBL)-negative strains—supporting its use as salvage therapy when guided by rapid diagnostics (Kuai et al., 2023). Fluoroquinolones also diverge; susceptible isolates maintain low ciprofloxacin resistance (≤11%), whereas CRPA sees significant increases (ciprofloxacin rising from about 25% to 31% and levofloxacin from 42% to 47%), highlighting the risks of sequential carbapenem–fluoroquinolone regimens that co-select resistance. Aminoglycosides and polymyxins remain treatment anchors. CRPA’s amikacin resistance stays under 11%, and polymyxin B generally preserves ≥81% susceptibility despite occasional spikes. These findings support combination strategies over monotherapy to curb further resistance. In sum, *P. aeruginosa*’s dual profiles demand agile, data-driven stewardship. Tailoring regimens—with options like amikacin plus ceftazidime/avibactam for MBL-negative CRPA and high-dose amikacin–aztreonam for MBL-positive cases—alongside real-time resistance monitoring is vital to outpace this organism’s adaptive evolution (Mikhail et al., 2019; Timsit et al., 2022).

The significant divergence in ceftazidime/avibactam resistance rates between our multicenter data (72.7% in 2023, n=22) and CHINET national surveillance (19.6% in 2023) necessitates a refined interpretation. While our study encompassed hospital-wide isolates, the predominance of tertiary care centers likely introduced systemic biases. Tertiary hospitals in Tianjin typically manage complex cases requiring prolonged broad-spectrum antimicrobial exposure and invasive interventions, fostering selective pressures that favor multidrug-resistant clones—a phenomenon less pronounced in CHINET’s broader hospital-level sampling. Methodological distinctions further clarify this gap. CHINET’s standardized protocols aggregate data across diverse care settings, whereas our cohort’s tertiary-heavy infrastructure may disproportionately represent antimicrobial “hotspots.”

Additionally, the limited 2023 CRPA sample size (n=22) amplifies stochastic variability, necessitating cautious extrapolation. Future work should stratify resistance patterns by care intensity to resolve these discrepancies. Targeted analysis of intensive care unit isolates within our 14-center cohort could validate whether resistance escalation correlates with care acuity—a critical hypothesis given that intensive care unit environments amplify antimicrobial selection pressures through concentrated β-lactam/carbapenem use. Such stratification would also clarify whether the observed 2023 resistance decline reflects true epidemiological shifts or transient sampling variance. These findings advocate for regional resistance surveillance systems tailored to local care hierarchies. While CHINET’s national data provide population-level benchmarks, our study highlights how region-specific hospital profiles (e.g., tertiary center density) can distort resistance landscapes, underscoring the need for granular, care-setting-specific stewardship strategies.

#### iv. A. baumannii resistance: enduring challenges, evolving solutions

The evolving resistance patterns of *A. baumannii* and CRAB in this study reveal encouraging trends and persistent challenges. The significant decline in aminoglycoside resistance—particularly gentamicin (33.9% to 28.9%) and amikacin (8.1% to 6.9%)—suggests the potential reversibility of resistance under optimized antimicrobial stewardship. This aligns with evidence that cyclic aminoglycoside “holidays” may reduce selective pressure in high-burden settings.

The observed resurgence of β-lactam/β-lactamase inhibitor combinations (BLICs) against *A. baumannii*—particularly ampicillin/sulbactam (36.2%→29.8% resistance) and piperacillin/tazobactam (44.0%→37.3%)—signals a paradigm shift in empiric therapy selection for non-CRAB infections. This trend mirrors findings from the MERINO-3 trial, where protocolized BLIC use reduced carbapenem consumption by 34% without compromising mortality in gram-negative bacteremia (Stewart et al., 2021). Clinicians should prioritize BLICs over carbapenems for non-severe *A. baumannii* infections in regions with resistance rates <40%, aligning with IDSA-SHEA antimicrobial stewardship guidelines (Barlam et al., 2016).

CRAB’s persistently bleak carbapenem susceptibility (imipenem: 99.1–99.6% resistance) necessitates aggressive combination regimens. Our findings substantiate the therapeutic promise of minocycline-polymyxin B combinations against CRAB, evidenced by declining minocycline resistance (55.5%→42.5%) and sustained polymyxin B susceptibility (≤7.7%). Mechanistically, polymyxin B potentiates minocycline by efflux pump disruption and enhanced intracellular accumulation, translating to synergistic lethality in murine pneumonia models (Bowers et al., 2015). However, rising tigecycline resistance (31.3%→37.0%) cautions against overreliance on tetracycline derivatives, especially given *A. baumannii’s* propensity for tet(X) gene-mediated efflux pump upregulation (Chen et al., 2020). Instead, emerging agents like cefiderocol—a siderophore cephalosporin achieving 63% CRAB susceptibility in the CREDIBLE-CR study (Bassetti et al., 2021)—should be integrated into salvage protocols where available.

The paradoxical fluoroquinolone resistance surge in CRAB (ciprofloxacin: 87.4–93.3%) despite global usage declines underscores the role of cross-resistance via gyrA mutations co-selected by cephalosporins (Morgan-Linnell et al., 2009). This phenomenon, previously documented in a 2023 whole-genome sequencing analysis of 1,200 CRAB isolates (Müller et al., 2023), mandates strict avoidance of fluoroquinolones in CRAB-endemic units. Instead, rapid diagnostic tools like the GenMark Dx ePlex^®^ BCID-GN panel should guide early targeted therapy, optimizing therapy compared with conventional methods (McCarty et al., 2023).

#### v. MRSA resistance evolution: precision management and future challenges

MRSA remains uniformly resistant to β-lactams—penicillin G resistance is ≥99% due to blaZ-mediated β-lactamase production—confirming that these agents are globally obsolete (Lade and Kim, 2023). In contrast, non-β-lactam options reveal mixed trends. Rifampicin resistance has significantly declined from 7.7% to 3.7%, likely reflecting stewardship measures restricting its monotherapy and promoting its use in biofilm-targeted combinations.

Meanwhile, resistance to fluoroquinolones and trimethoprim-sulfamethoxazole is rising. Levofloxacin resistance increased from 20.2% to 30.7%, driven by accumulating grlA mutations and NorA overexpression, while trimethoprim-sulfamethoxazole resistance climbed from 8.9% to 13.6%, pointing to potential dfrA gene spread in community strains (Hooper, 2000; Ambrose and Hall, 2021). Additionally, gentamicin resistance jumped from 10.6% to 20.5%, likely due to the clonal spread of aminoglycoside-modifying enzymes, which undermines its role in combination regimens for endocarditis (Garneau-Tsodikova and Labby, 2016).

In stark contrast, linezolid, vancomycin, and teicoplanin maintain 100% susceptibility. Vancomycin’s enduring efficacy is secured through optimized AUC/MIC (Area Under the Curve to Minimum Inhibitory Concentration) dosing, and linezolid’s stability reflects careful stewardship (Men et al., 2016). However, emerging concerns—such as vancomycin-intermediate *Staphylococcus aureus* and cfr(chloramphenicol-florfenicol resistance)-mediated linezolid resistance—underscore the need for proactive molecular surveillance (Weinstein and Fridkin, 2001; Morales et al., 2010; Pfaller et al., 2017).

Clinically, these findings advocate for a recalibrated therapeutic hierarchy. For invasive MRSA infections, vancomycin or teicoplanin should serve as the backbone—with linezolid reserved for central nervous system involvement or vancomycin intolerance—while rifampicin can be leveraged as a synergistic adjunct in prosthetic joint infections when used with bactericidal agents. Given the rising resistance, fluoroquinolones and trimethoprim-sulfamethoxazole should be limited to culture-guided outpatient therapy, and gentamicin may retain niche utility in endocarditis combinations with rigorous monitoring.

#### vi. Enterococcal infections: divergent profiles and targeted therapy

Our data reveal divergent resistance trends between *Enterococcus faecalis* and *Enterococcus faecium*. *E. faecalis* maintains high ampicillin susceptibility (≤2.8% resistance), reflecting its infrequent acquisition of β-lactamase genes (Murray, 1990). In stark contrast, *E. faecium* exhibits ≥88% ampicillin resistance driven by PBP5 mutations, highlighting its hospital-adapted genomic plasticity (Galloway-Peña et al., 2011; Montealegre et al., 2017).

Fluoroquinolone resistance is rising in *E. faecalis* (ciprofloxacin ~32–35%; levofloxacin ~30–35%), but in *E. faecium* it reaches near-pan-resistance (≥90%), mandating exclusion from empiric therapy. Linezolid resistance in *E. faecalis* increased modestly from 5.6% to 7.5%, whereas in *E. faecium*, resistance remained low and relatively stable (1.0% ~ 2.4%), underscoring the importance of culture-directed therapy and molecular screening. Both species demonstrate high-level aminoglycoside resistance (gentamicin ~36–39% in *E. faecalis* and ~32–35% in *E. faecium*), limiting their synergistic role in severe infections and necessitating therapeutic drug monitoring. Streptomycin resistance further narrows options, favoring alternatives like daptomycin in high-resistance scenarios. Glycopeptides remain reliable, with vancomycin and teicoplanin resistance staying below 0.9% in both species, though sporadic teicoplanin resistance calls for ongoing vigilance.

Clinically, *E. faecalis* infections can typically be managed with ampicillin (or amoxicillin), reserving glycopeptides for beta-lactam allergic or multidrug-resistant cases, while *E. faecium* requires glycopeptides or daptomycin—with linezolid limited to confirmed Vancomycin-resistant *Enterococcus* cases (O'Driscoll and Crank, 2015). Empiric fluoroquinolones should be avoided, favoring nitrofurantoin or fosfomycin for urinary tract infections. Rapid molecular diagnostics (e.g., vanA/B, cfr PCR) and therapeutic drug monitoring are essential to optimize therapy and reduce toxicity (Miller et al., 2014). With few new agents on the horizon, preserving existing options through precision prescribing and integrated resistance surveillance is critical to outpacing these adaptable pathogens.

#### vii. Regional resistance divergence: reconciling local and national surveillance

Antimicrobial resistance patterns identified in our multicenter cohort in Tianjin revealed substantial deviations from national surveillance benchmarks reported by CHINET between 2018 and 2022 (Yang et al., 2023). Specifically, our data demonstrated that our MRSA isolates exhibited near-complete resistance to β-lactams and increasing non-susceptibility to levofloxacin, TMP-SMX, and gentamicin—findings that stand in contrast to CHINET’s documented downward trends for these agents. Likewise, Enterococcus faecalis and E. faecium in our cohort displayed extensive multidrug resistance, with E. faecalis notably harboring a broader spectrum of resistance and virulence determinants than typically captured in national datasets.

Among Gram-negative organisms, resistance profiles in our Escherichia coli isolates were characterized by high rates of non-susceptibility to ampicillin, fluoroquinolones, and third-generation cephalosporins, largely attributable to the rising prevalence of ESBL producers. This contrasts with CHINET’s surveillance, which reported modest declines in resistance to ceftriaxone and carbapenems. Our Klebsiella pneumoniae isolates showed near-universal resistance to ampicillin and a concerning upward trend in carbapenem non-susceptibility—diverging from CHINET’s relatively stable carbapenem resistance and declining rates for agents such as ciprofloxacin and ceftazidime-avibactam. For Pseudomonas aeruginosa, our findings indicated elevated resistance to carbapenems and fluoroquinolones, whereas CHINET data suggested a downward trajectory across most antipseudomonal agents. Most notably, Acinetobacter baumannii in our cohort exhibited widespread resistance to β-lactams, carbapenems, and fluoroquinolones, with only marginal susceptibility to last-line agents such as colistin and tigecycline—again, in contrast to the more stable resistance patterns reported nationally.

Several factors likely account for the observed divergence in resistance profiles. Our cohort, sourced primarily from tertiary care centers, comprises patients with increased exposure to invasive procedures, prolonged hospitalization, and intensive use of broad-spectrum antimicrobials—conditions that amplify selective pressures favoring resistant phenotypes, including metallo-β-lactamase–producing *P. aeruginosa*. The specimen composition further complicates interpretation: a disproportionately high number of sputum samples in our dataset enriched for respiratory pathogens with intrinsically elevated resistance, whereas CHINET’s broader sampling scope may dilute such signals. Institutional prescribing patterns also play a role; frequent use of carbapenems and fluoroquinolones in high-acuity settings likely co-selects for resistance mechanisms such as *oprD* porin loss and efflux pump overexpression, contributing to inflated local resistance metrics. Methodological considerations must also be acknowledged. Although both datasets adhere to CLSI standards, decentralized testing in our study may introduce subtle inter-laboratory variability.

Addressing these discrepancies requires a more granular approach to resistance surveillance. Future efforts should stratify data by hospital tier, care intensity, and specimen type to disentangle true epidemiological trends from sampling bias. Incorporating ICU-specific analyses and molecular characterization of resistance determinants (e.g., *bla_VIM*, *bla_IMP*, *bla_NDM*) will be essential for contextualizing phenotypic resistance within its genetic framework. Ultimately, regional surveillance systems must evolve to reflect institutional heterogeneity, ensuring that antimicrobial stewardship strategies are not only pathogen-directed but structurally aligned with the realities of clinical care.

### Limitations and future directions

This study has several important limitations. First, the absence of molecular resistance profiling—such as PCR detection of carbapenemase genes (*bla_KPC*, *bla_NDM*, *bla_OXA*) or whole-genome sequencing—limits mechanistic interpretation of key trends, including the observed improvement in ceftazidime-avibactam susceptibility among CRPA isolates. Future studies should incorporate molecular diagnostics to clarify clonal dynamics and resistance gene dissemination.

Second, the statistical analysis relied primarily on p-values, without reporting effect sizes, confidence intervals, or corrections for multiple comparisons. Given the volume of resistance metrics evaluated, more rigorous statistical frameworks are needed to enhance interpretability and reduce false positives.

Third, the retrospective design introduces selection bias and lacks standardized clinical metadata (e.g., demographics, comorbidities, prior antibiotic exposure), constraining the clinical relevance of resistance patterns. Prospective studies with harmonized data collection would enable subgroup analyses and risk stratification.

Fourth, although the cohort includes 13 tertiary and one secondary hospital, resistance data were not stratified by hospital level. Institutional differences in patient acuity, stewardship practices, and infection control may significantly influence resistance dynamics. Future research should incorporate hospital-level stratification to assess these effects.

Lastly, comparisons with national platforms such as CHINET are limited by structural and methodological disparities. Our tertiary-heavy sample may overrepresent high-risk populations, while CHINET’s broader sampling may dilute resistance signals. Stratified regional surveillance frameworks are needed to reconcile these differences and support precision stewardship.

## Conclusion

This multicenter retrospective study offers a comprehensive overview of microbial distribution and antimicrobial resistance across 14 hospitals in Tianjin from 2021 to 2023. Findings highlight a growing predominance of Gram-negative pathogens—particularly *Klebsiella pneumoniae*—and a notable shift toward respiratory specimens. While carbapenems and aminoglycosides retain efficacy against select organisms such as *E. coli* and *P. aeruginosa*, rising resistance to cefepime, tigecycline, and fluoroquinolones calls for reassessment of empirical regimens. Encouraging reversals in susceptibility to ceftazidime-avibactam and cefotetan suggest potential alternatives for managing carbapenem-resistant infections.

However, limitations—including the absence of molecular profiling, lack of clinical metadata, and unstratified hospital-level analysis—underscore the need for more granular, mechanistically informed surveillance. Future studies should adopt prospective designs, integrate rapid molecular diagnostics, and stratify data by care intensity and institutional tier to better inform clinical decision-making. Ultimately, precision antimicrobial stewardship must move beyond descriptive epidemiology. By combining real-time resistance analytics with molecular insights and contextualized care data, targeted interventions can be developed to curb resistance and optimize outcomes across diverse healthcare settings.

## Data Availability

The raw data supporting the conclusions of this article will be made available by the authors, without undue reservation.
