# Supplementary material for: Navigating an evolving microbial landscape: emerging antimicrobial resistance trends and precision stewardship in Tianjin tertiary hospitals (2021–2023)
Source: Front Cell Infect Microbiol. 2025 Sep 8;15:1629038. doi: 10.3389/fcimb.2025.1629038 (PMC12450913; doi:10.3389/fcimb.2025.1629038)
Supplement: Supplementary file 1 [file DataSheet1.zip › supplementary material/Supplementary Table Captions.docx]

**Supplementary Table Captions**

Table S1. Distribution of isolates by sample source and composition ratio.

Table S2. Top five bacterial isolates and their proportions

Table S3. Top five gram-positive bacterial isolates and their proportions

Table S4. Top five gram-negative bacterial isolates and their proportions

Table S5. Top five Enterobacteriaceae isolates and their proportions

Table S6. Top five non-fermentative bacterial isolates and their proportions

Table S7. Resistance rates of *Escherichia coli* to commonly used antibiotics

Tables S8. Resistance rates of *Klebsiella pneumoniae* to commonly used antibiotics

Table S9. AMR rates of CRKP to common antibiotics

Table S10. AMR rates of *Pseudomonas aeruginosa* to common antibiotics

Table S11. AMR rates of CRPA to common antibiotics

Table S12. AMR rates of *Acinetobacter baumannii* to common antibiotics

Table S13. AMR rates of CRAB to common antibiotics

Table S14. AMR rates of methicillin-resistant *Staphylococcus aureus* (MRSA) to common antibiotics

Table S15. AMR rates of *Enterococcus faecalis* to common antibiotics

Table S16. AMR rates of *Enterococcus faecium* to common antibiotics
